# Characterization of the Metabolic, Clinical and Neuropsychological Phenotype of Female Carriers of the Premutation in the X-Linked *FMR1* Gene

**DOI:** 10.3389/fmolb.2020.578640

**Published:** 2020-10-22

**Authors:** Eleonora Napoli, Yingratana Amabel McLennan, Andrea Schneider, Flora Tassone, Randi J. Hagerman, Cecilia Giulivi

**Affiliations:** ^1^Department of Molecular Biosciences, School of Veterinary Medicine, University of California, Davis, Davis, CA, United States; ^2^MIND Institute, University of California Davis Medical Center, Sacramento, CA, United States; ^3^Department of Pediatrics, University of California Davis Medical Center, Sacramento, CA, United States; ^4^Department of Biochemistry and Molecular Medicine, School of Medicine, University of California, Davis, Davis, CA, United States

**Keywords:** mitochondrial dysfunction, omics, cellular response to stress, oxidative phosphorylation, glycolysis, fragile X-associated primary ovarian insufficiency, fragile X-associated tremor and ataxia syndrome

## Abstract

The X-linked *FMR1* premutation (PM) is characterized by a 55–200 CGG triplet expansion in the 5′-untranslated region (UTR). Carriers of the PM were originally thought to be asymptomatic; however, they may present general neuropsychiatric manifestations including learning disabilities, depression and anxiety, among others. With age, both sexes may also develop the neurodegenerative disease fragile X-associated tremor/ataxia syndrome (FXTAS). Among carriers, females are at higher risk for developing immune disorders, hypertension, seizures, endocrine disorders and chronic pain, among others. Some female carriers younger than 40 years old may develop fragile X-associated primary ovarian insufficiency (FXPOI). To date, no studies have addressed the metabolic footprint – that includes mitochondrial metabolism – of female carriers and its link to clinical/cognitive manifestations. To this end, we performed a comprehensive biochemical assessment of 42 female carriers (24–70 years old) compared to sex-matched non-carriers. By applying a multivariable correlation matrix, a generalized bioenergetics impairment was correlated with diagnoses of the PM, FXTAS and its severity, FXPOI and anxiety. Intellectual deficits were strongly correlated with both mitochondrial dysfunction and with CGG repeat length. A combined multi-omics approach identified a down-regulation of RNA and mRNA metabolism, translation, carbon and protein metabolism, unfolded protein response, and up-regulation of glycolysis and antioxidant response. The suboptimal activation of the unfolded protein response (UPR) and endoplasmic-reticulum-associated protein degradation (ERAD) response challenges and further compromises the PM genetic background to withstand other, more severe forms of stress. Mechanistically, some of the deficits were linked to an altered protein expression due to decreased protein translation, but others seemed secondary to oxidative stress originated from the accumulation of either toxic mRNA or RAN-derived protein products or as a result of a direct toxicity of accumulated metabolites from deficiencies in critical enzymes.

## Introduction

Carriers of the premutation (PM) are characterized by a moderate (55 to 200) expansion of the cytosine-guanine-guanine (CGG) nucleotide repeats in the first exon and promoter of the X-linked *FMR1* gene (Verkerk et al., 1991; Bagni et al., 2012). Originally, PM carriers were assumed to be free of any apparent phenotypic traits. However, over the last decade, a growing number of neuropsychiatric manifestations (including depression, anxiety, and insomnia), visuospatial deficits, and immune dysregulation have been reported to occur at a greater frequency among adult PM carriers than in the general population (Hagerman and Hagerman, 2013). Generally, PM carriers show lower performance in neuropsychological testing including full-scale intellectual quotient (FSIQ) and working memory (WM) subtests on the Wechsler Adult Intelligence Scale (Lozano et al., 2016). In the case of children with the PM, they are often diagnosed with ADHD, autism, anxiety, and other psychopathologies (Napoli et al., 2018). The PM has also been associated with conditions beyond those involving the CNS, such as hypertension, hypothyroidism, high blood glucose, as well as a higher incidence of thyroid, prostate and other cancers (Lozano et al., 2016). With age, both female and male carriers of the PM are at a higher risk for developing the late-onset (usually appearing after age 50) neurodegenerative disorder fragile X-associated tremor/ataxia syndrome [FXTAS; OMIM:300623; (Hagerman et al., 2001, 2004; Hagerman and Hagerman, 2013)]. FXTAS-affected carriers may exhibit intention tremor and gait ataxia, accompanied by cerebral atrophy, white matter disease, parkinsonism, neuropathy, autonomic dysfunction, and cognitive deficits (Berry-Kravis et al., 2007a; Bourgeois et al., 2007; Lozano et al., 2016; Claeys et al., 2020).

As it is the case for many X-linked disorders, women have a lower absolute risk of developing FXTAS symptoms compared to men (Hagerman and Hagerman, 2016). However, women carrying *FMR1* PM allele have a higher risk of developing premature or primary ovarian insufficiency (POI) [16% (Schwartz et al., 1994; Allingham-Hawkins et al., 1999; Rifé et al., 2004; Terracciano et al., 2004; Schuettler et al., 2011; Elizur et al., 2014; Pouresmaeili and Fazeli, 2014)] as compared to full mutation females (>200 CGG repeats), who carry the same risk for POI as the general population (1%). About 20 to 30% of female carriers experiencing irregular periods or amenorrhea due to ovarian insufficiency prior to age 40 are diagnosed with fragile X-associated primary ovarian insufficiency [FXPOI; (Allingham-Hawkins et al., 1999; Allen et al., 2007)]. Even PM carriers without signs of ovarian dysfunction have an earlier (on average by 5 years) age at menopause compared with non-carriers (Patsalis et al., 1999; Sullivan et al., 2005; Besterman et al., 2014). Women with alleles between 35–44 CGG repeats seem to present diminished ovarian function but regular menses and occult primary ovarian insufficiency (Streuli et al., 2009; Karimov et al., 2011; Pastore et al., 2012); however, other studies found no association between *FMR1* intermediate alleles and POI (Bennett et al., 2010; Murray et al., 2014; Voorhuis et al., 2014). Other medical and psychological issues reported in females are hypothyroidism, hypertension, endocrine dysfunctions, chronic pain, fibromyalgia, autoimmune diseases, neuropathies, migraines, dementia, and psychiatric conditions, such as anxiety and depression (Allen et al., 2007, 2020; Bailey et al., 2008; Hunter et al., 2010; Winarni et al., 2012; Wheeler et al., 2014a,b; Lozano et al., 2016; Movaghar et al., 2019). Collectively included under the term FXAND [fragile X–associated neuropsychiatric disorders; (Hagerman et al., 2018)], such emotional and neuropsychiatric disorders, have been shown to be more common in female carriers compared to non-carriers. In a recent work by Dr. S. Sherman’s group, which investigated the association between the PM diagnosis and CGG repeat expansion in female carriers, the most common symptoms reported were anxiety and depression, migraine, headaches, and sleep problems (Allen et al., 2020).

Our team was the first to report mitochondrial dysfunction as a common feature in biological samples from PM carriers (Napoli et al., 2013, 2016a,b, 2018; Song et al., 2016) as well as in murine models of the PM (Napoli et al., 2016a). This decreased mitochondrial bioenergetics is present in PM carriers with and without FXTAS and even in some pediatric carriers (Napoli et al., 2018). However, to our knowledge, no study has to date characterized the metabolic footprint of the PM and related clinical and cognitive features in female carriers of the PM.

To bridge this gap in knowledge, we performed a comprehensive biochemical assessment (including metabolomics and proteomics profiling, and bioenergetics) in peripheral blood mononuclear cells (PBMC) and plasma samples obtained from 24- to 70-year-old female carriers. To elucidate peripheral bioenergetics markers that may function as surrogates for CNS function, we utilized a multivariable correlation matrix to identify correlations between mitochondrial outcomes and cognitive parameters (FSIQ), executive function (BDS-2), anxiety, tremor, and FXTAS (and its severity) and FXPOI diagnoses. As such, this study is ideally positioned to perform comprehensive deep metabolic and mitochondrial phenotyping by gathering complementary outcomes on genomics, proteomics, metabolomics, mitochondrial physiology and clinical information to systematically develop a metabolic profile from established female carriers by taking advantage of a substantial repository of patient samples. This multi-faceted approach, as opposed to a simple model based on the statistical differences of few (and sometimes unconnected) metabolites or proteins between diagnostic groups. The profile generated will, for the first time, allow researchers to fully assess the larger biological impact of the premutation on metabolic status of female carriers, thereby providing unprecedented insight into the biological consequences of metabolic deficits and mitochondrial dysfunction, aiding the discovery of disease mechanisms. Importantly, due to the depth of the phenotyping across multiple readouts, the integrated metabolic profiles generated will have the detail required to cluster patients according to their clinical pathology when more patients’ data will be available. This necessary step, in turn, will lead to truly novel and testable hypotheses regarding individualized pathogenesis and treatment.

## Materials and Methods

### Subjects

Blood samples were obtained from 42 female carriers of the *FMR1* PM ranging from 24- to 70-year old, recruited through the Fragile X Treatment and Research Center at the MIND Institute at University of California, Davis. Blood samples were also obtained from 10 female non-carriers aged 25 to 60 years. The study was approved by the IRB ethics committee at University of California Davis Medical Center. Blood samples were obtained by venipuncture with informed consent. FXTAS was diagnosed utilizing criteria reported before (Jacquemont et al., 2003; Hagerman and Hagerman, 2016). For returning participants, outcomes evaluated at one single visit collected at the indicated age were included in the analysis.

### Genotyping

CGG repeat expansion in all individuals included in this study were evaluated by both PCR and Southern Blot analysis, as previously described (Tassone et al., 2008; Filipovic-Sadic et al., 2010). The X-activation ratio (XAR), representing the percentage of cells with the normal allele on the active X chromosome, was calculated by the ratio of the densitometric intensity of the normal *FMR1* unmethylated band over the sum of the intensities of the normal unmethylated and methylated bands (Tassone et al., 1999; Berry-Kravis et al., 2003). In a population of normal (Z) distribution, 1.65 × SD leads to a tail that gives the probability of 5% of the data to be excluded from normal. If this value is subtracted from the mean XAR, then anything below this value has <5% probability of being significant. Thus, XAR values <36% of non-carrier ones were considered unfavorable (<0.2).

### Lymphocyte Preparation

Blood (5–7 ml) was collected in BD vacutainer CPT tubes (BD Biosciences, Franklin Lakes, NJ, United States) and lymphocytes were isolated as previously described (Napoli et al., 2016a). Upon collection, lymphocyte suspension was divided into 2 aliquots in Eppendorf tubes and pelleted by centrifugation 1 min 2,000 rpm in a microfuge at 4°C. The supernatant was removed, and the pellet was used immediately for mitochondrial outcomes. An aliquot of PBMC was suspended in 0.5 ml cold 10 mM HEPES, pH 7.4, frozen at −80°C overnight and subsequently transferred for extended storage, into liquid nitrogen.

### Mitochondrial Outcomes

All chemicals and biochemicals were of analytical grade or higher. Enzymatic activities of Complexes I–V in digitonin-permeabilized lymphocytes determined by polarography essentially as described before (Giulivi et al., 2010; Napoli et al., 2016a). Briefly, an aliquot (0.5–1.0 × 10^6^) of lymphocytes was added to the oxygen chamber in 0.3 ml of a buffer containing 0.22 M sucrose, 50 mM KCl, 1 mM EDTA, 10 mM KH_2_PO_4_, and 10 mM HEPES, pH 7.4. Oxygen consumption rates were evaluated in the presence of (i) 1 mM ADP plus 1 mM malate-10 mM glutamate followed by the addition of 5 μM rotenone; (ii) 10 mM succinate followed by the addition of 1 mM malonate; (iii) 1 mM α-glycerophosphate followed by the addition of 3.6 μM antimycin A; and (iv) 10 mM ascorbate and 0.2 mM *N,N,N*′*,N*′*-*tetramethyl-*p-*phenylenediamine followed by the addition of 1 mM KCN. Activities of individual electron transport chain (ETC) segments were evaluated as the difference of oxygen uptake recorded before and after the addition of specific inhibitors. Citrate synthase activity was evaluated spectrophotometrically with a Tecan Infinite M200 microplate reader equipped with the Magellan software (Austria) at 412 nm as described elsewhere (Napoli et al., 2016a). The respiratory control ratio (RCR) was calculated as the ratio between oxygen uptake rates of intact cells supplemented with 10 mM glucose (present in RPMI-1640) in State 3 μ (with 2 μM carbonylcyanide-*p*-trifluoromethoxyphenylhydrazone, or FCCP) and State 4 (with 0.2 μM oligomycin) (Giulivi et al., 2013). Mitochondrial ROS production was calculated from the oligomycin-resistant oxygen consumption rates and normalized by basal respiration in the presence of 10 mM glucose.

### Plasma Metabolomics

Plasma samples were obtained from age-matched 8 non-carriers and 7 PM carriers as previously described (Napoli et al., 2016a), and metabolites were extracted and analyzed by mass spectrometry (Napoli et al., 2015). Briefly, 30-μl aliquots were extracted by 1 ml of degassed acetonitrile:isopropanol:water (3:3:2, V/V/V) at −20°C, centrifuged and decanted with subsequent evaporation of the solvent to complete dryness. A clean-up step with acetonitrile/water (1:1) removed membrane lipids and triglycerides. Details on the identification of metabolites and data analysis are reported in Napoli et al. (2015).

### Proteomics

Peripheral blood mononuclear cells samples were hand-homogenized on ice in 20 mM HEPES pH 6.8, containing protease and phosphatase inhibitor cocktail (Sigma-Aldrich, St. Louis, MO, United States). Protein concentration was determined by BCA protein assay kit (Thermo Scientific, Sunnyvale, CA, United States) and samples from 7 controls and 7 PM carriers were submitted to the UC Davis Mass Spectrometry Facility and analyzed as described in detail elsewhere (Napoli et al., 2016a).

### Statistics

As the population assessed in this study did not follow a normal distribution, Spearman’s correlation coefficients and correspondent *p* values for each pair of variables tested were computed with a correlation matrix for multiple variables. Due to the limited information available for some subjects on some of the variables included in our analysis (Table 1), and to exclude the possibility of considering significant interactions without true biological relevance, we applied a more stringent cut-off conditions by setting *p* values at ≤0.01. We did not employ a Bonferroni correction to adjust the *p* values for the number of variables tested as (i) it ignores dependencies among the data and is therefore too conservative if the number of tests is large, (ii) it assumes that all null hypotheses are true simultaneously, which was not our assumption, (iii) it increases the likelihood of type II errors, so that truly important differences are considered non-significant (Perneger, 1998). A two-way ANOVA was carried out to test for the contribution of age, diagnosis and age x diagnosis on the mitochondrial outcomes’ variability. Graph Pad Prism v. 8.1.2 was used for all the statistical analyses. Metabolomics and proteomics data were analyzed as previously described (Giulivi et al., 2016; Napoli et al., 2016a,b).

**TABLE 1:** Demographic and clinical data of the women included in this study.

*Clinical group*	*Age (y)*	*CGG*	*XAR*	*FXTAS Stage*	*FXPOI*	*FSIQ*	*BDS2*	*Anx*
C1*	25	29,30	ND	0	No	115	18	No
C2*	26	24,33	ND	0	No	ND	ND	ND
*C3*	27	40,42	ND	0	No	121	ND	ND
C4*^¶^	29	20,33	ND	0	No	106	26	No
C5*	33	30,37	ND	0	No	121	26	No
C6*	44	23,30	ND	0	No	104	ND	No
C7*	45	22,33	ND	0	No	ND	ND	No
C8*	47	23,30	ND	0	No	ND	24	No
*C9*	54	30	ND	0	No	ND	ND	No
C10*^¶^	60	23,30	ND	0	No	133	25	Yes
P1*^¶^	24	30,79	0.78	0	No	125	24	No
P2*	24	31,93	0.38	0	No	96	19	No
P3*	33	30,137	0.55	0	No	96	22	Yes
*P4*	33	29,81	0.57	0	No	ND	ND	ND
*P5*	37	30,79	0.72	0	No	ND	ND	ND
P6*(T)	38	33,60	0.43	0	No	112	24	No
*P7*	38	43,78	0.15	0	No	ND	ND	ND
P8*(T)	43	30,106	0.55	0	No	98	22	No
P9*	49	31,86	0.64	0	Yes	118	25	Yes
P10*	50	20,98	0.88	0	No	123	26	No
*P11*	50	30,94	0.56	0	ND	ND	ND	ND
*P12*	50	22,119	0.68	0	No	90	20	Yes
*P13*^¶^	52	29,81	0.68	0	No	105	23	No
*P14*^¶^	56	30,69	0.42	0	Yes	114	26	Yes
*P15*^¶^	57	30,68	0.40	0	ND	ND	ND	ND
*P16*	58	29,69	0.57	0	No	ND	23	No
*P17*	58	27,77	0.48	0	No	98	23	Yes
*P18*	60	30,84	0.90	0	No	ND	ND	ND
*P19*	60	23,87	0.86	0	Yes	ND	ND	ND
*P20*	62	30,84	0.15	0	No	100	26	Yes
*P21*	64	31,71	0.49	0	No	ND	23	Yes
*P22*	71	29,105,160	0.81	0	Yes	110	23	Yes
*F1*	54	31,102	0.68	2.5	Yes	104	25	Yes
*F2*	54	32,93	0.62	3	ND	ND	ND	ND
*F3*	56	30,93	0.15	3	HYS	ND	10	Yes
*F4*	57	30,99	0.76	2	No	96	21	Yes
*F5*	59	33,107	0.10	ND	No	ND	ND	ND
*F6*	60	37,70	0.62	3	No	ND	ND	ND
*F7*	60	31,100	0.53	3	No	ND	ND	ND
*F8*	62	37,107	0.57	2	No	131	25	No
*F9*	63	30,102	0.36	3	HYS	104	22	Yes
*F10*	64	30,82	0.60	4	No	92	23	Yes
*F11*	67	23,103	0.31	3	No	104	21	Yes
*F12*	68	23,88	0.92	3	ND	ND	ND	ND
*F13*	68	25,57	0.66	3	Yes	106	26	No
*F14*	68	30,74	0.68	3	Yes	115	21	Yes
*F15*	70	28,104	0.77	4	Yes	97	17	No
*F16*	70	20,85	0.52	2	No	103	19	Yes
*F17*	70	29,61	0.85	2	ND	ND	ND	ND
*F18*	70	30,110	0.42	3	No	ND	ND	ND
*F19*	70	29,105	0.64	2	HYS	105	17	Yes
*F20*	70	30,76	0.54	3	Yes	99	ND	Yes

* Metabolomics carried out in plasma. *^¶^* Proteomics carried out in PBMC. Anx, anxiety; BDS2, behavioral dyscontrol scale II; FSIQ, Full Scale IQ, calculated by Wechsler Adult Intelligence Scale, Fourth Edition (WAIS-IV) P and F indicate, respectively, PM carriers either without or with FXTAS; FXTAS stage refers to clinical evaluation determined by previous diagnosis and based on the score published in Jacquemont et al. (2003) and Hagerman et al. (2008). HYS indicates patients that underwent hysterectomy (F3 at 27 year; F9 at 40 year; F19 at 36 year). T indicates the presence of tremors. ND, not determined or determined but not yet available in the database.

## Results

### Demographics and Clinical Characteristics of the Cohorts

Our cohorts consisted of women with the PM with and without FXTAS, and sex-matched non-carriers (named hereafter controls; Table 1). The control study group consisted of 25–60 year old women with CGG repeats at the 5′- untranslated region (UTR) of *FMR1* of 31 ± 1 (mean ± SEM; *n* = 10). The PM group included 24- to 70-year-old females (*n* = 42), from which about half (*n* = 20) were diagnosed with FXTAS. The FXTAS stages ranged from 1 to 4, with most subjects at stage 3. The CGG repeats of the mutant allele ranged from 57 to 137, with an average of 87 ± 4 for PM carriers without FXTAS and 90 ± 4 for FXTAS-affected carriers with no significant difference between these two groups (*p* = 0.156). Of the 42 carriers, 10 were diagnosed with FXPOI, and 3 had undergone hysterectomy between the age of 26 and 40 years. XAR ranged from 0.1 to 0.92 with an average of 0.57 ± 0.04 in PM without FXTAS and 0.57 ± 0.05 in FXTAS-affected with no significant difference between these two groups (*p* = 0.878), and not different from the expected random inactivation of the X-chromosome. Only 4 of 42 carriers had an unfavorable XAR.

Both cohorts were assessed for most of the neurological/neuropsychiatric outcomes. They included full-scale intellectual quotient (FSIQ; Table 1) assessed with the Wechsler Adult Intelligence Scale, WAIS-IV (Drozdick et al., 2012), and executive function [assessed by the Behavioral Dyscontrol Scale II or BDS-2; (Belanger et al., 2005)]. Subjects were also tested for generalized anxiety through the Structured Clinical Interview for Diagnosis of Mental Disorders (SCID)-1 [Diagnostic and Statistical Manual of Mental Disorders (DSM)-IV; Table 1].

### Deficits in Peripheral Mitochondrial Energy Production Are Linked to Cognitive and Psychiatric Outcomes, and Diagnoses of FXPOI and FXTAS and Its Morbidity (FXTAS Stage)

**FIGURE 1:**
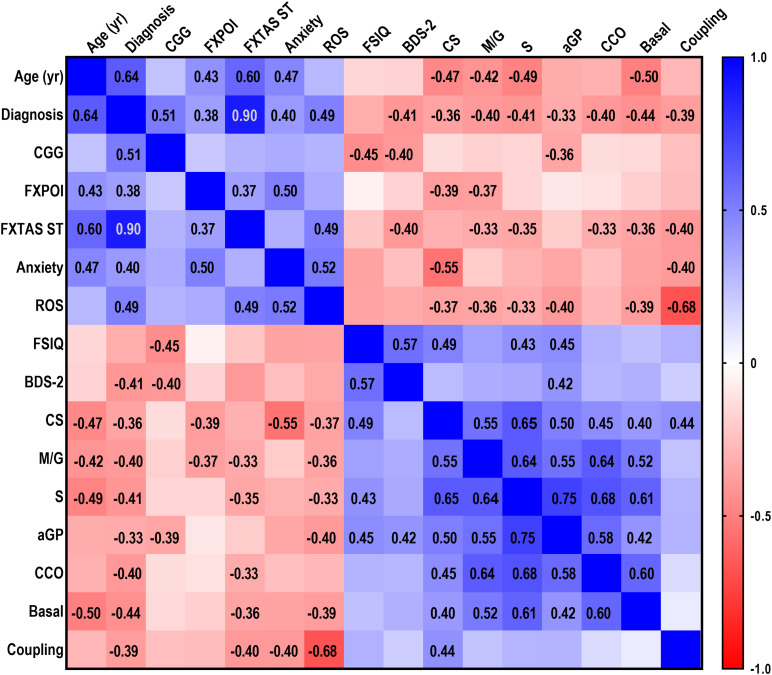
Correlations between demographic, clinical and mitochondrial outcomes. A multivariable correlation matrix was built with demographic, clinical and functional data relative to PM without (*n* = 22) and with (*n* = 20) FXTAS symptoms and control (*n* = 10) women age 24–70 year, along with biochemical mitochondrial outcomes measured in lymphocytes obtained from the same individuals. Outcomes analyzed were: age, diagnosis, CGG (all with *n* = 52), FXPOI (*n* = 47), FXTAS stage (*n* = 51), anxiety (*n* = 36), Full Scale IQ (FSIQ; *n* = 31), Behavioral Dyscontrol Scale-2 (BDS-2; *n* = 32), ROS (*n* = 46), citrate synthase activity (CS; *n* = 52), NADH-fueled ATP-linked O_2_ consumption (M/G; *n* = 52), succinate (S)-sustained FADH_2_-fueled ATP-linked linked O_2_ consumption (*n* = 52), α-glycerophosphate (αGP)-dependent ATP-linked O_2_ consumption (*n* = 50), cytochrome *c* oxidase activity (CCO; 51), basal respiration (*n* = 51), coupling (*n* = 50). Categorical variables (i.e., diagnosis, FXPOI, anxiety) were assigned a numerical value. Diagnosis: control = 0, PM without FXTAS = 1, PM with FXTAS = 2; FXPOI and anxiety: absence = 0, presence = 1. Due to the non-Gaussian distribution of the data, the non-parametric Spearman test was run. *R* values are shown for those correlations which were statistically significant at *p* ≤ 0.01. A scale showing the range of *r* values (from –1.0 for inversely correlated outcomes to 1.0 for positively correlated ones) is also shown.

A multivariable correlation matrix was built by utilizing as input data clinical, psychiatric and molecular outcomes from non-carriers and PM carriers (Figure 1). Continuous variables were used as such, whereas categorical ones (e.g., three diagnoses: non-carriers, PM, FXTAS; FXPOI diagnosis, and presence of anxiety) were assigned numerical values. Tremor, one of FXTAS hallmarks, was excluded from the correlation matrix as it was available for 23 of the 42 participants, of which only 2 showed tremors. Similarly, the XAR was not shown in Figure 1, as this outcome did not show any correlation with any of the other reported outcomes, due to the low frequency (9.5%) of unfavorable (<0.2) XAR in our cohort. The apparent discrepancy with the findings of other studies (Hagerman and Hagerman, 2004; Berry-Kravis et al., 2005, 2007b) could be bridged by considering that they utilized other neurological features (Berry-Kravis et al., 2007b); the use of an updated clinical diagnostic criteria for FXTAS (Hagerman and Hagerman, 2013).

Overall, demographic (age), clinical (diagnoses of PM and FXTAS, CGG repeats, diagnosis of FXPOI and FXTAS stage) and psychiatric (anxiety) variables correlated positively with each other. Notably, all of these outcomes were associated with increased oxidative stress as they were directly correlated with mitochondrial ROS production. As expected, these outcomes were inversely correlated with intellectual and functional capacities (FSIQ and BDS-2) and mitochondrial function (Figure 1).

In order to narrow down the significant associations among variables, the non-parametric Spearman’s correlation coefficients, and correspondent *p* values were calculated.

#### Diagnoses of PM, FXTAS and FXPOI

Using the above mentioned significance threshold, FXTAS and PM diagnoses significantly and inversely correlated with (i) activity of citrate synthase, marker of mitochondrial mass, (CS; Spearman’s *r* = −0.361, *p* = 0.004); (ii) mitochondrial ATP production supported by NADH- (*r* = −0.397, *p* = 0.001) and FADH_2_-linked substrates (*r* = −0.413, *p* = 0.001); (iii) α-glycerophosphate-fueled ATP production (αGP; *r* = −0.330, *p* = 0.01); (iv) cytochrome *c* oxidase activity (*r* = −0.402, *p* = 0.001); (v) glucose-sustained mitochondrial ATP production (*r* = −0.439, *p* < 0.0001); and (vi) coupling between electron transport and ATP production (*r* = −0.391, *p* = 0.002). In turn, diagnoses directly correlated with mitochondrial ROS production (*r* = 0.490, *p* < 0.0001). The same biochemical outcomes, except for mitochondrial mass (CS activity) and αGP-mediated respiration, correlated with FXTAS severity (i.e., stage; Figure 1) indicating that the morbidity of this neurodegenerative disease is also reflected as a peripheral deficit in bioenergetics.

Lower CS activity and NADH-linked ATP production characterized FXPOI, diagnosis strongly linked to the occurrence of anxiety (*r* = 0.497, *p* = 0.001; Figure 1). While PM and FXTAS diagnoses, morbidity of FXTAS and FXPOI were all inversely correlated with CS activity, it could be assumed that the lower bioenergetics was a result of lower mitochondrial mass. However, even after normalization of the rates of ATP production by mitochondrial mass, several outcomes were still correlated (some directly and others inversely) highlighting the delicate balance among Complexes within the electron transport chain that needs to be preserved for its adequate production of ATP while minimizing ROS production. Among them (normalized by mitochondrial mass), NADH-linked ATP production with anxiety (*r* = 0.469; *p* = 0.002); succinate-linked ATP production with anxiety and PRI (*r* = 0.370; *p* = 0.01); CCO activity with FXPOI diagnosis (*r* = 0.310; *p* = 0.02), anxiety (*r* = 0.407; *p* = 0.008), PRI (*r* = −0.565; *p* = 0.01) and FSIQ (*r* = −0.369; *p* = 0.02); and basal respiration with PRI (*r* = −0.653; *p* = 0.003) and PSI (*r* = −0.517; *p* = 0.02).

#### Anxiety

Anxiety was inversely correlated with CS activity (*r* = −0.553, *p* < 0.0001) and coupling (*r* = −0.398, *p* = 0.01), and directly with mitochondrial ROS production (*r* = 0.521, *p* = 0.001).

### Cognition and Executive Function

FSIQ strongly and positively correlated with executive function (BDS-2; *r* = 0.566, *p* < 0.0001; Figure 1). Similarly, and considering that the brain represents the main site of energy consumption with over 20% of body’s total oxygen consumption (Watts et al., 2018), a strong and direct correlation was identified between FSIQ and overall mitochondrial bioenergetics (Figure 1), with statistically significant associations with mitochondrial mass (CS, *r* = 0.489, *p* = 0.002), FADH_2_-linked ATP production (*r* = 0.427, *p* = 0.009), and α-glycerophosphate-fueled ATP production (αGP; *r* = 0.446, *p* = 0.008). Overall, BDS-2 positively correlated with mitochondrial function, with the correlation with α-glycerophosphate-fueled ATP production resulting statistically significant (*r* = 0.417, *p* = 0.008). [NADH- and FADH_2_-linked ATP production were significantly correlated with BDS-2 at *p* < 0.05 with *r* = 0.318 and 0.337, respectively].

#### FMR1 Gene Structure

No correlation was obtained between CGG expansions and FXPOI diagnosis likely due to the significant number of carriers with >100 CGG repeats (28.6%) as reported by others (Sullivan et al., 2005; Ennis et al., 2006; Allen et al., 2007). However, CGG repeats showed a statistically significant inverse correlation with α-glycerophosphate-fueled ATP production (*r* = −0.391, *p* = 0.009), probably indicating issues with the redox state and regulation of cellular energy metabolism, as well as with FSIQ (*r* = −0.448, *p* = 0.008) and BDS-2 performance (*r* = −0.400, *p* = 0.01).

#### Age

A strong and inverse correlation was observed between age and mitochondrial function in both carriers and non-carriers (Figure 1) consistent with the age-dependent decline of OXPHOS capacity (Burch et al., 1963; Cardellach et al., 1989; Linnane et al., 1989; Yen et al., 1989; Byrne et al., 1991; Cooper et al., 1992; Müller-Höcker, 1992; Torii et al., 1992; Münscher et al., 1993; Boffoli et al., 1994, 1996; Lezza et al., 1994; Wallace et al., 1995; Capkova et al., 2002) being more evident in tissues with high OXPHOS demand (Wallace, 1992; Ojaimi et al., 1999; Kwong and Sohal, 2000; Quiles et al., 2002). Relevant to our study, the overall age-dependent decline in the OXPHOS capacity is more evident in women (Cooper et al., 1992; Papa, 1996). To discriminate between the contribution of age- and the PM-dependent decline in mitochondrial function, we carried out a two-way ANOVA analysis for each mitochondrial outcome evaluated in PM and non-carriers at two age ranges, i.e., younger (23–43 year) and older (44–60) (Table 2). Then the same analysis was done with PM and FXTAS-affected females at two age ranges, i.e., younger (50–60 year) and older (60–70 year), to test for the contribution of age and FXTAS diagnosis (Table 2). When comparing controls and PM carriers, a simple main effect analysis showed that NADH- and FADH_2_-linked ATP production along with the activity of CCO were solely attributed to the carrier status. Only CS activity showed a decline dependent with both age and PM status (Table 2). When the same analysis was performed to compare mitochondrial outcomes between PM and FXTAS-affected females, FXTAS diagnosis affected significantly both mtROS production and coupling, whereas all other outcomes were not influenced either by age or diagnosis. Of note, the observed correlation between age and FXPOI does not reflect the age at which these carriers first experienced ovarian insufficiency, but merely the age at which the PM carriers reported their medical history.

**TABLE 2:** Effect of diagnosis, age and diagnosis x age interaction on mitochondrial outcomes evaluated in controls, PM and FXTAS female carriers.

	Diagnosis x age interaction	Diagnosis effect	Age effect
**Controls vs. PM**			
ROS (DFn = 1, DFd = 26)	*F* = 1.05e-003	*F* = 1.45	*F* = 0.01
	*p* = 0.9744	*p* = 0.2396	*p* = 0.9133
CS (DF*n* = 1, DFd = 24)	*F* = 0.57	*F* = 4.84	*F* = 6.83
	*p* = 0.4560	***p* = 0.0377**	***p* = 0.0153**
M/G (DFn = 1, DFd = 26)	*F* = 0.07	*F* = 4.75	*F* = 0.46
	*p* = 0.7989	***p* = 0.0385**	*p* = 0.5019
S (DFn = 1, DFd = 25)	*F* = 0.21	*F* = 4.80	*F* = 0.08
	*p* = 0.6542	***p* = 0.0381**	*p* = 0.7863
αGP (DFn = 1, DFd = 26)	*F* = 0.04	*F* = 8.08	*F* = 0.09
	*p* = 0.8433	***p* = 0.0086**	*p* = 0.7623
CCO (DFn = 1, DFd = 26)	*F* = 1.8e-003	*F* = 6.82	*F* = 0.02
	*p* = 0.9665	***p* = 0.0148**	*p* = 0.9026
Basal (DFn = 1, DFd = 27)	*F* = 0.01	*F* = 5.94	*F* = 4.16e-005
	*p* = 0.9362	***p* = 0.0217**	*p* = 0.9949
Coupling (DFn = 1, DFd = 26)	*F* = 0.08	*F* = 1.23	*F* = 1.23
	*p* = 0.7832	*p* = 0.2760	*p* = 0.4929
**PM vs. FXTAS**			
ROS (DFn = 1, DFd = 35)	*F* = 0.36	*F* = 9.36	*F* = 0.42
	*p* = 0.5537	***p* = 0.0042**	*p* = 0.5230
CS (DFn = 1, DFd = 41)	*F* = 2.20	*F* = 0.50	*F* = 1.67
	*p* = 0.1461	*p* = 0.4839	*p* = 0.2029
M/G (DFn = 1, DFd = 40)	*F* = 2.42	*F* = 0.28	*F* = 1.89
	*p* = 0.1279	*p* = 0.5971	*p* = 0.1766
S (DFn = 1, DFd = 39)	*F* = 1.24e-004	*F* = 3.48	*F* = 0.05
	*p* = 0.9912	*p* = 0.0697	*p* = 0.8193
αGP (DFn = 1, DFd = 39)	*F* = 0.09	*F* = 0.11	*F* = 0.11
	*p* = 0.7604	*p* = 0.7376	*p* = 0.7408
CCO (DFn = 1, DFd = 41)	*F* = 0.55	*F* = 2.54	*F* = 0.26
	*p* = 0.4632	*p* = 0.1189	*p* = 0.6161
Basal (DFn = 1, DFd = 40)	*F* = 0.40	*F* = 0.65	*F* = 0.55
	*p* = 0.5296	*p* = 0.4242	*p* < 0.4632
Coupling (DFn = 1, DFd = 40)	*F* = 1.71e-003	*F* = 5.00	*F* = 0.14
	*p* = 0.9672	***p* = 0.0312**	*p* = 0.7118

*αGP, alpha-glycerophosphate; Basal, glucose-sustained mitochondrial ATP production; CCO, cytochrome *c* oxidase; CS, citrate synthase activity; M/G, malate/glutamate; ROS, reactive oxygen species; S, succinate. DF, degrees of freedom; DFn, number of groups -1; DFd, number of subjects – DFn. Numerosity of the groups is as follows. For the comparison between controls and PM: younger controls, *n* = 4; older controls *n* = 4; younger PM, *n* = 7, older PM, *n* = 13. For the comparison between younger and older carriers: younger PM, *n* = 17; older PM, *n* = 5; younger FXTAS, *n* = 5; older FXTAS, *n* = 15). Note: in some instances, outcomes for 1–6 samples were not tested for lack of biological material. Bolded are statistically significant p values (<0.05).*

### RNA and Protein Metabolism, Glycolysis and Cellular Response to Stress Is Differentially Regulated in Females With PM

**FIGURE 2:**
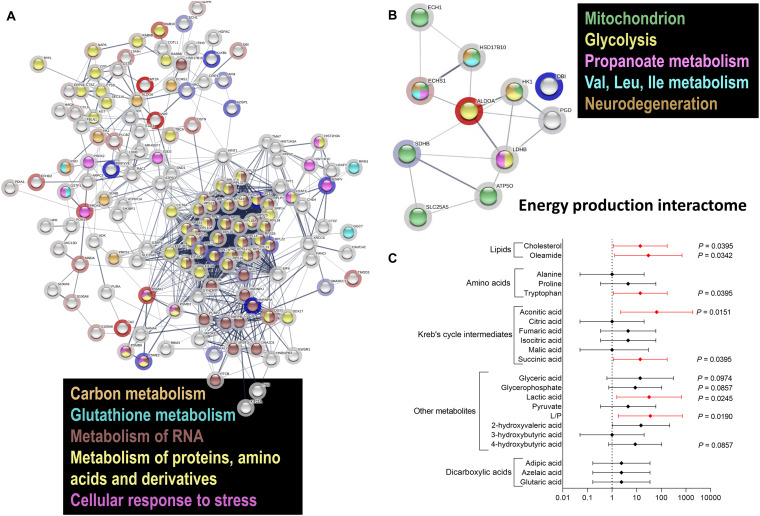
Differential protein and metabolite enrichment in PM females. **(A)** Proteomics analysis was carried out in PBMC from PM females 29–63 year old and age-matched controls. Proteins detected in both PM and non-carriers were uploaded with their corresponding fold change values (see Supplementary Dataset) to STRING. Interactomes were algorithmically generated based on direct associations (physical or functional) between eligible proteins. The interactomes are color coded with blue nodes representing proteins that were down-regulated, red upregulated in PM, and clear when there were detected but whose levels were not statistically different between diagnostic groups. The shading of each node is correlated with the magnitude of the fold change. Differentially regulated metabolic pathways included carbon metabolism, metabolism of RNA, proteins, amino acids, and cellular response to oxidative stress. **(B)** A subset of differentially regulated proteins (Supplementary Dataset, tabs highlighted in gray) had key roles in mitochondrial function, glycolysis, fatty acid and amino acid metabolism, as well as in RNA processing (pathways reported in Supplementary Figures 1–3) and pathways affected in neurodegeneration. **(C)** Metabolomics analysis was performed in plasma from 8 controls and 7 age-matched PM carriers, 24–52 year, and age-matched controls. Differentially enriched metabolites are shown, with their respective *p* values, in Supplementary Figure 4. A forest plot was built with the odds ratios (*X* axis) and the 95% CI (error bars) calculated with control values for each selected metabolite based on its role in glycolysis and mitochondrial metabolism. In red are metabolites with statistically significant OR, indicating a higher probability to be affected in the PM, and as such considered as putative biomarkers for female carriers.

Considering that impaired energy metabolism in the brain is integral to many CNS diseases, cells display complex proteomic responses to energy deficits, including activation of UPR and limited protein synthesis, among others. To gain a deeper insight on the molecular mechanisms of the responses originating from the impaired energy deficit in a PM genetic background, we utilized a proteomics approach on PBMCs from a subset of carriers (see demographics details of this subset under Table 1). Proteins significantly altered between PM and non-carriers were uploaded with their corresponding fold change values (Supplementary Dataset) to STRING (Szklarczyk et al., 2019). Interactomes were algorithmically generated based on direct associations (physical or functional) between eligible proteins. STRING generated a score for each interactome which is a putative measure of probability, with the shading of each node being positively correlated with the magnitude of the fold change. The main functional interactome showed upregulation of the following pathways: HIF-1 signaling, glycolysis, sugars and carbon metabolism, pentose phosphate pathway, pathways involved in cancer-related faulty transcriptional regulation, and pathways affected in Alzheimer’s disease (Table 3). Among the downregulated ones were ribosomal-, spliceosomal- (Supplementary Figure 1) and proteasomal-related pathways (Table 3; Supplementary Figure 2; Supplementary Dataset), pyruvate metabolism, amino acids degradation (in particular branched amino acids), RNA processing (Table 3; Supplementary Figure 2; Supplementary Dataset) and transport, fatty acid synthesis, and TCA (Table 3; Figure 2A; KEGG and REACTOME Pathway tabs of Supplementary Dataset). This interactome highlighted a shift in mitochondrial energy production (and consistent with the previous results on bioenergetics), enhanced endoplasmic reticulum (ER) stress and ribosomal dysfunction. A sub-interactome (named “Energy production interactome”) was linked to energy-producing pathways including mitochondrial bioenergetics and glycolysis (Figure 2B; Supplementary Figure 3; Supplementary Dataset, tabs highlighted in gray). The decrease in proteins involved in butanoate metabolism, fatty acid elongation and beta-oxidation along with BCAA metabolism (ECH1, HSD17B10, and ECHS1) indicates a shift toward glycolysis (higher HK1, ALDOA, but lower LDHB) as alternative energy source without incurring into increased ketone bodies’ metabolism.

**TABLE 3:** Differential expression of pathways in PBMC from PM females.

Upregulated	Raw p
HIF-1 signaling pathway	0.0011698
Glycolysis or Gluconeogenesis	0.013441
Fructose and mannose metabolism	0.02419
Neomycin, kanamycin and gentamicin biosynthesis	0.036192
Central carbon metabolism in cancer	0.091013
Bacterial invasion of epithelial cells	0.10011
Alzheimer disease	0.13034
Transcriptional misregulation in cancer	0.15592
Pentose phosphate pathway	0.19871

**Downregulated**	**Raw p**

Ribosome	1.24E-17
Spliceosome	0.016999
Propanoate metabolism	0.025582
Pyruvate metabolism	0.033013
Proteasome	0.042895
Valine, leucine and isoleucine degradation	0.0482
Thermogenesis	0.089012
Non-homologous end-joining	0.091438
Complement and coagulation cascades	0.1142
RNA transport	0.12057
Fatty acid elongation	0.18073
Butanoate metabolism	0.18677
Citrate cycle (TCA cycle)	0.19871

*For the detailed list of proteins identified by proteomics analysis see Supplementary Dataset. Proteomics analysis was evaluated in 2 controls and 3 PM without FXTAS (Table 1).*

Since the protein changes provided insight into the complex molecular alterations underpinning the cellular adaptation to the PM-mediated stress, we sought to evaluate circulating metabolites and biomarkers of mitochondrial dysfunction by performing untargeted plasma metabolomics. To this end, untargeted metabolomics was performed by using gas chromatography mass spectrometry in plasma from 7 carriers and 8 age-matched controls (see demographics details under Table 1). Similar to the metabolic disarray observed in some mitochondrial disorders, Krebs’ cycle intermediates, dicarboxylic acids, and “other metabolites” associated with glycolysis and leucine metabolism were mostly up-regulated (Supplementary Figure 4), with levels of aconitate, succinate, lactic acid, and lactate-to-pyruvate, being significantly higher in PM than controls (Supplementary Figure 4). These results confirm and expand findings of increased Krebs’ cycle intermediates, likely the result of an impaired TCA activity, already observed by our group in PM carriers of both sexes (Giulivi et al., 2016), with aconitate, and isocitrate being those that differentiated the most between diagnostic groups, along with oleamide (Giulivi et al., 2016). In this regard, levels of oleamide, along with tryptophan, were found significantly lower in female PM whereas those of cholesterol were significantly higher in PM (Supplementary Figure 4). The odds ratio (OR) for these metabolites to be altered in the PM (Figure 2C with *p* < 0.1) was significant for cholesterol, oleamide, tryptophan, aconitate, succinate, lactate, and lactate-to-pyruvate ratio, glycerate, alpha-glycerophosphate, 4-hydroxybutyrate and some of the dicarboxylic acids tested.

## Discussion

Mounting evidence has shown that female carriers of the PM are at higher risk for developing several health-related issues compared to non-carrier females (Bailey et al., 2008; Hunter et al., 2010; Winarni et al., 2012; Wheeler et al., 2014b; Movaghar et al., 2019). Awareness of these risks and correlation of the clinical signs with the biochemical footprint of carriers could help to identify critical biomarkers in early diagnosis and likely prognosis. If ascertained, these associations could lead to an integrated approach between clinical specialties and basic science, which could be extremely beneficial in the management of symptoms and challenges that female PM carriers experience in their day-to-day life.

The brain’s high mitochondrial energy consumption makes neurons highly vulnerable to impaired glucose metabolism (Hyder et al., 2013). Then, it is not surprising that a decline in mitochondrial activity has been associated with memory loss and, particularly, with age-dependent cognitive impairment (Freeman and Young, 2000; Liu et al., 2002). Moreover, most mtDNA diseases are associated with brain disorders because adequate neuronal development (Atamna et al., 2002; Zini et al., 2002) and structure (Johnson and Byerly, 1993; Bristow et al., 2002; Liu et al., 2002) and axonal and synaptic activity (Freeman and Young, 2000; Zenisek and Matthews, 2000) all involve mitochondrial genes (Wallace, 1999; Schon, 2000). Mitochondrial dysfunction has been reported not only in neurodegeneration (Friedland et al., 1983; Minoshima et al., 1997; Chiaravalloti et al., 2015) but also in pre-symptomatic, genetically-susceptible individuals (Small et al., 1995; Reiman et al., 1996; Mosconi et al., 2006; Ossenkoppele et al., 2013). In line with these studies and our previous reports on the PM (Napoli et al., 2016a, 2018), our study strongly indicates that global impairment of bioenergetics, and likely the subsequent energy depletion, is one of the earliest functional changes prior to the onset of overt clinical symptoms. This is supported by the relatively milder mitochondrial dysfunction in PM carriers without FXTAS and FXPOI which is enhanced with the diagnosis and progression of FXTAS. While the decline in cognitive/intellectual (FSIQ) and executive (BDS-2) function was correlated with longer CGGs, intellectual decline (FSIQ) was significantly correlated with an overall mitochondrial deficit. This is highly suggestive that mitochondrial deficits may influence neuronal dysregulation and, over the years, degenerative mechanisms such as described for AD (Rice et al., 2014).

The metabolomics findings are particularly relevant in the context of several psychiatric and neurological symptoms experienced by some female carriers as oleamide has a critical role in mood and sleep disorders as well as depression due to its interaction with serotonergic and GABAergic neurotransmission (Mendelson and Basile, 2001). Similarly, tryptophan is not only an essential amino acid for protein synthesis but also a precursor of several biological mediators involved in stress response, antioxidant system, behavioral response, and immune system (Firk and Markus, 2009; Hoglund et al., 2017). Decreased tryptophan levels have been linked to lower serotonin levels, likely setting the basis for the establishment of neuropsychological symptoms including depression, anxiety, irritability, attention deficits, and insomnia (Jenkins et al., 2016). The higher cholesterol levels are of interest in the context of neurodegeneration, as mitochondrial cholesterol loading has recently emerged as a key player in the pathology of neurological disorders such as AD and Niemann-Pick Type C (NPC) disease (Elustondo et al., 2017; Torres et al., 2019).

**FIGURE 3:**
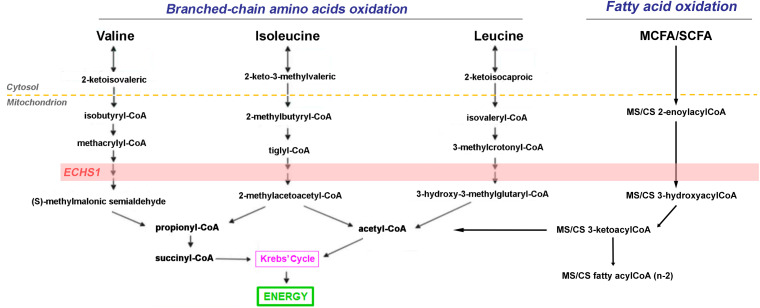
Role of ECSH1 in branched-chain amino acids and short- and medium-chain fatty acid catabolism. The short-chain enoyl-CoA hydratase ECHS1 has a critical role in branched chain amino acid (BCAA) catabolism as well as fatty acid catabolism generating succinyl-CoA and acetyl-CoA which are fed into the Krebs’ cycle for the generation of reducing equivalents.

In line with these findings, the lower fatty acid beta-oxidation resulting mainly from lower ABAD and ECHS1, deserves a separate discussion. Lower levels of ABAD may disrupt the ABAD-beta-amyloid interaction in mitochondria and suppresses apoptosis by increasing the levels of beta-amyloid as reported for AD patients and transgenic mouse models (Lustbader et al., 2004). Deficiency in ECHS1 results in metabolic acidosis with a combined respiratory chain deficiency (Sakai et al., 2015). Consistent with these findings, higher levels of lactic acid and a trend towards higher 2-hydroxyvaleric acid, proline and organic acids, such as adipic, azelaic and glutaric, were observed in plasma from female carriers (Figures 2C, 3) similar to the cases reported by others (Fitzsimons et al., 2018). Then, the significant lower expression of ABAD and ECHS1 in female carriers makes them as interesting candidates to investigate further in terms of its regulation in the context of beta-amyloid and proteostasis. Furthermore, since the OXPHOS decline in ECHS1 deficiency has been attributed to the accumulation of inhibitory fatty acid intermediates and to the disruption of ETC Complex biogenesis and/or stability (Burgin and McKenzie, 2020), it would not be unlikely that these mechanisms are contributing to the OXPHOS deficiencies observed in the PM.

Proteomics analysis not only confirmed and extended the functional and metabolomics results, but also shed light into the proteostasis status of the PM. The biological consequence of higher mitochondrial ROS was identified with the higher levels of aconitate likely the result of inactivation of aconitase, an enzyme highly sensitive to oxidative stress damage. The increase in oxidative stress may contribute to proteotoxicity and to the generation of misfolded proteins that accumulate upon ER stress, as a result, increase in the detoxification demands. However, an uncoordinated proteomic response in the ER was identified in the PM. Only one chaperone, HSPA5, was higher in PM vs. non-carriers, and a mixed response was observed for subunits of the proteasome. These results suggest that proteasomal degradation, a fundamental mechanism for degrading the misfolded proteins that accumulate in response to the metabolic challenge, is somehow not coordinated to adequately cope with the proteotoxicity. Indeed, it has been a long-standing hypothesis that protein aggregates in diseased brain impair the protein degradation function of the 26S proteasome (Taylor et al., 2002; Ciechanover and Brundin, 2003; Valera et al., 2005), then it would be reasonable to hypothesize that as the UPR is not fully coordinated in female carriers, any proteasomal response expected to ensue in response to a metabolic, environmental or pharmacological challenge might be adversely affected.

Our findings are particularly relevant for female carriers, as mitochondria have a critical role in oocyte developmental competence and function (Dumollard et al., 2007; Wang et al., 2009), and in the development and function of the reproductive system fertility (Reynier et al., 2001; El Shourbagy et al., 2006; Santos et al., 2006; Cagnone et al., 2016; Demain et al., 2017) with an irrefutable implication in primary ovarian insufficiency (POI) (Conca Dioguardi et al., 2016; Tiosano et al., 2019). The energy deficit along with the lower levels of ECHS1 in carriers, one of the genes identified as critical for oocyte developmental competence (Biase, 2017), set the basis for future research to identify more clearly the role of this protein in POI and FXPOI.

In conclusion, through a combined multi-omics approach in PBMC and plasma of PM females in association with thorough clinical and bioenergetics assessments, we found impaired metabolic pathways which can result from the direct action of toxic intermediates derived from the PM genetic background (either accumulation of mRNA or proteotoxicity of RAN-derived protein products). In this regard, we are adding the novel observation that the accumulation of aberrant metabolites resulting from deficiencies in critical metabolic steps may add to the altered interaction between fatty acid oxidation and the electron transport chain contributing to the overall OXPHOS decline as it has been described for ECHS1 deficiency (Burgin and McKenzie, 2020; Figure 3). In turn, either mechanism elicits a suboptimal activation of UPR and ERAD responses, setting a challenging scenario to withstand other, more severe forms of stress. Along these lines, the development of neurodegeneration or other clinical symptoms in older carriers could be linked to a lifetime accumulation of cellular damage, aggravated by the aging process.

### Limitations

There are potential limitations to our study, starting with its partly retrospective design. Of all the participants included, neurocognitive testing and omics analyses were performed in a subset of individuals. The selection of the cohort was from a controlled sample of individuals enrolled in the study upon referral to the medical center clinic. For this reason, this cohort might not be representative of the full PM population range but rather the most affected ones. Similarly, a fraction of the controls included in our analysis were family members of probands with typical *FMR1* repeat sizes and, while they may represent the non-carrier group, they may be affected by extra emotional burden by having a relative or partner with PM diagnosis. The challenging recruitment of healthy donors is reflected in their relatively lower number particularly in the 60–70 year age bracket. Information on anxiety was self-reported. The OXPHOS variability could be ascribed to the lack of a clinical diagnosis of a mitochondrial disease (i.e., borderline OXPHOS capacity without overt mitochondrial disease symptoms) which is manifested in front of a challenge (high-intensity exercise, a co-morbidity, or aging), the influence of different dietary habits and lifestyle, and the contribution of mtDNA polymorphisms to cognitive deficits (Skuder et al., 1995).

## Financial Disclosure

RH has received funding from the Azrieli Foundation, Zynerba, Ovid and Neuren for treatment trials in fragile X syndrome, and consulted with Zynerba and Fulcrum regarding treatment for fragile X syndrome. FT has received funding from the Azrieli Foundation and Zynerba for studies on fragile X syndrome and has consulted with Zynerba. The other authors have no financial disclosures relevant to this article.

## Data Availability Statement

The raw data supporting the conclusions of this article will be made available by the authors, without undue reservation.

## Ethics Statement

The studies involving human participants were reviewed and approved by IRB Ethics Committee at UC Davis Medical Center. The patients/participants provided their written informed consent to participate in this study.

## Author Contributions

EN processed all samples, carried out all polarographic and spectrophotometric measurements, performed correspondent statistical analyses, wrote the manuscript, and revised and approved the final version as submitted. YM collected and provided demographic, clinical, and molecular data, revised the manuscript, and approved the final version as submitted. AS carried out the neuropsychological testing and also revised the manuscript and approved the final as submitted. FT provided CGG repeats and XAR, revised the manuscript, and approved the final version as submitted. RH carried out clinical assessment of the women enrolled in this study and wrote clinical findings, revised the manuscript, and approved the final manuscript as submitted. CG conceptualized and designed the study, analyzed the omics data, wrote the manuscript, and approved the final manuscript as submitted.

## Conflict of Interest

The authors declare that the research was conducted in the absence of any commercial or financial relationships that could be construed as a potential conflict of interest.
