# Supplementary material for: Characterization of the Metabolic, Clinical and Neuropsychological Phenotype of Female Carriers of the Premutation in the X-Linked FMR1 Gene
Source: Front Mol Biosci. 2020 Oct 22;7:578640. doi: 10.3389/fmolb.2020.578640 (PMC7642626; doi:10.3389/fmolb.2020.578640)
Supplement: Supplementary file 2 [file Data_Sheet_1.PDF]

Supplementary Figures for "Characterization of the metabolic, clinical and neuropsychological phenotype of female carriers of the premutation in the X-linked FMR1 gene" by Napoli *et al*.

Supplementary Figure 1 Differentially regulated proteins involved in UPR and proteasome degradation in female carriers
Proteomics analysis was carried out in PBMC from PM females, 29-63 y old, and age-matched controls. Proteins involved in unfolded protein response (UPR) and proteasome degradation pathways that were found upregulated in PM females are highlighted in red, downregulated ones are highlighted in blue. Details about fold-change for each target are reported in **Supplementary Dataset**.

Supplementary Figure 2 Differentially regulated proteins involved in mRNA processing and ribosomes in PM female carriers
A general downregulation (highlighted in blue) of proteins involved in mRNA processing and ribosomal activity was recorded in PM female carriers. See **Supplementary Dataset** for details on fold-change and correspondent *p* values.

Supplementary Figure 3 Differentially regulated proteins with critical roles in glycolysis and OXPHOS in the PM
Among the most differentially regulated proteins are: HK1 (Hexokinase 1), enzyme presiding the phosphorylation of glucose to glucose-6-phosphate; LDHB (Lactate Dehydrogenase B), which catalyzes the interconversion of pyruvate and lactate with concomitant interconversion of NADH and NAD^+ in a post-glycolysis process; SDHB (succinate dehydrogenase complex Fe-S subunit B), one of the subunits of succinate dehydrogenase (SDH, or Complex II), involved in Krebs' cycle and ETC; ATP5O oligomycin-sensitive subunit of the mitochondrial ATPase. In red are upregulated proteins, in blue are negatively regulated ones. See **Supplementary Dataset** for details on fold-changes and correspondent *p* values.

Supplementary Figure 4 Differential levels of metabolites in plasma from PM females
Top 22 metabolites identified as differentially accumulated in plasma from PM carriers relative to controls. In blue are LOG2FC for each metabolite. In orange are *p* values, shown as negative LOG p-value.

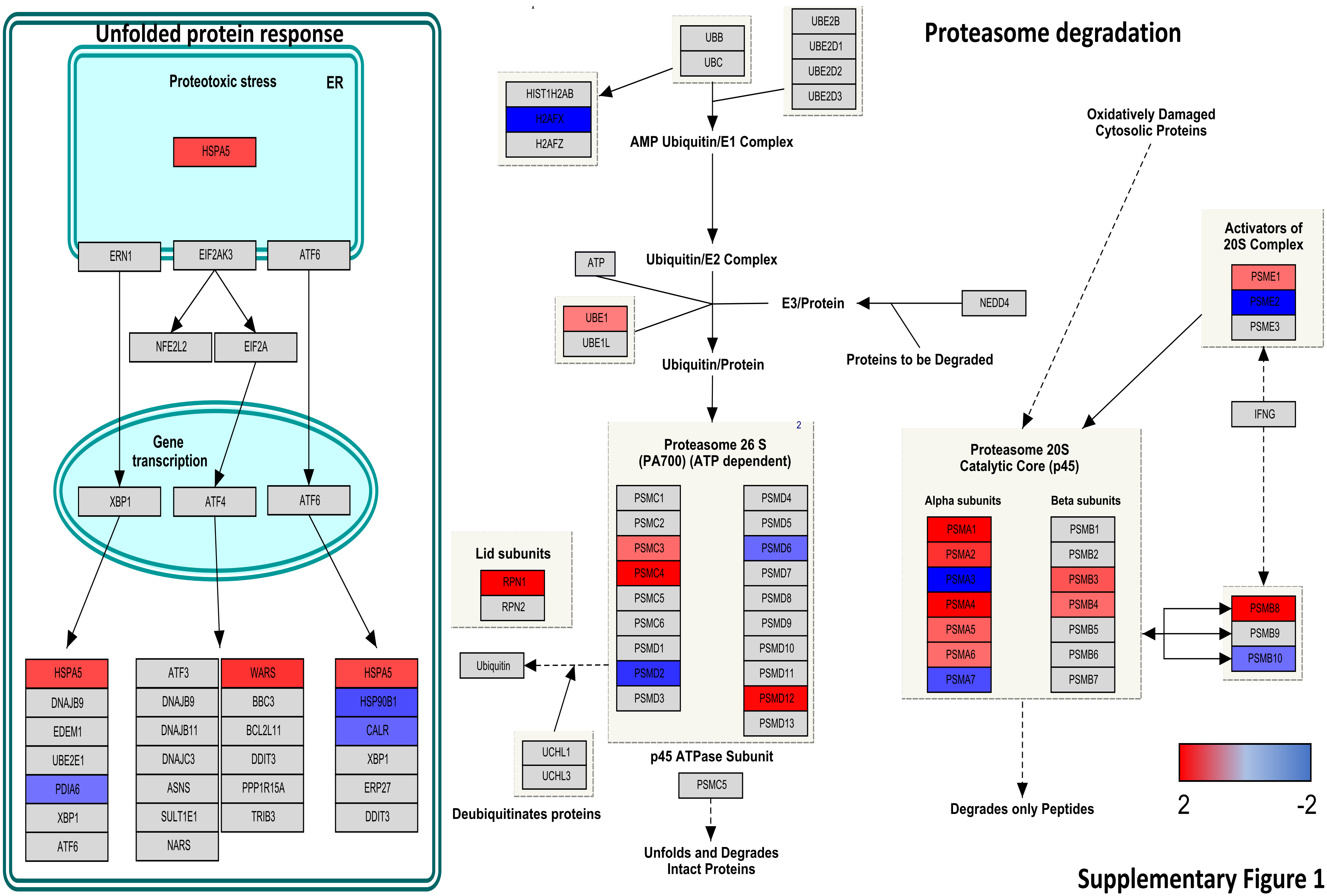

Supplementary Figure 1

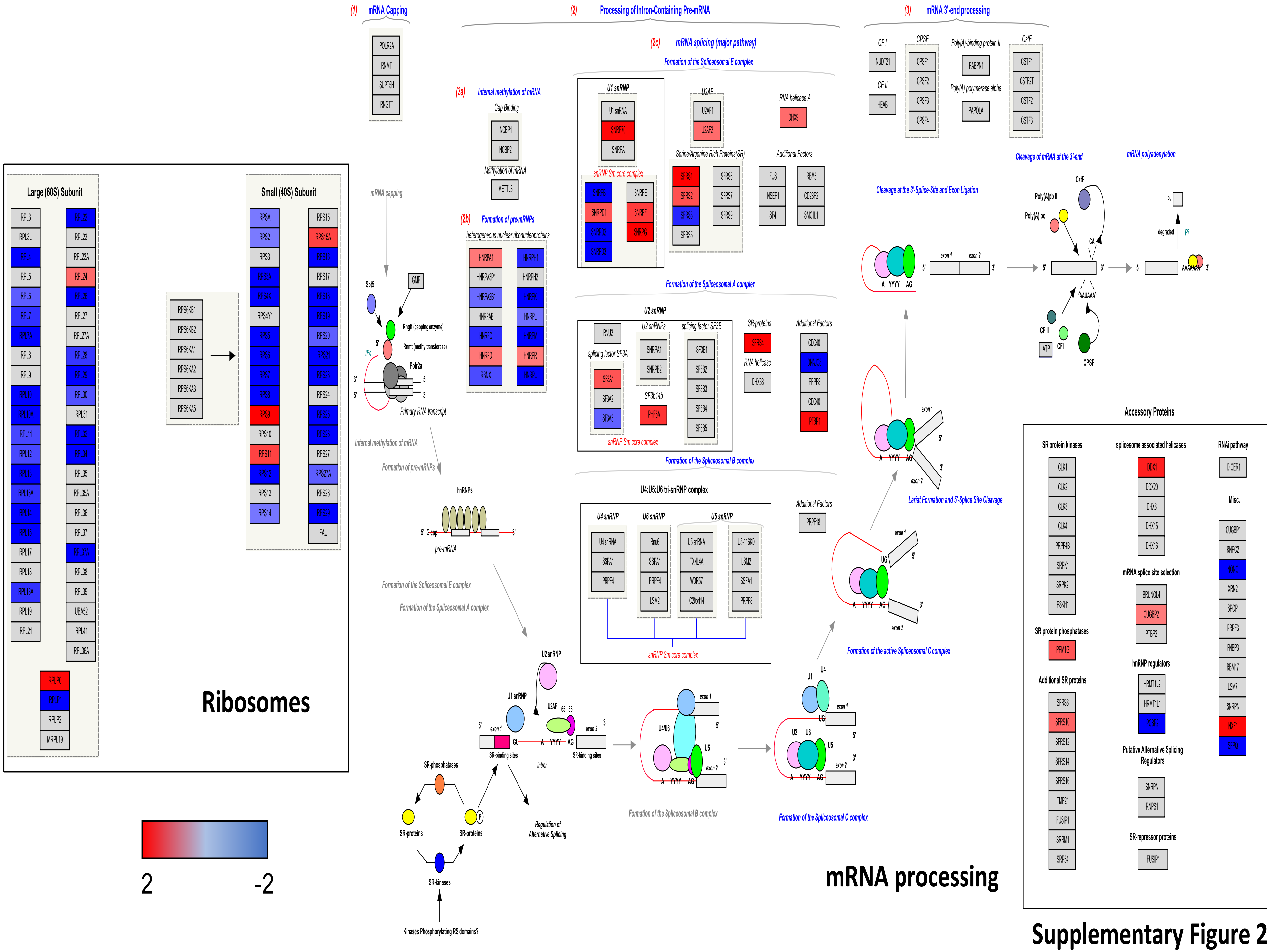

Supplementary Figure 2

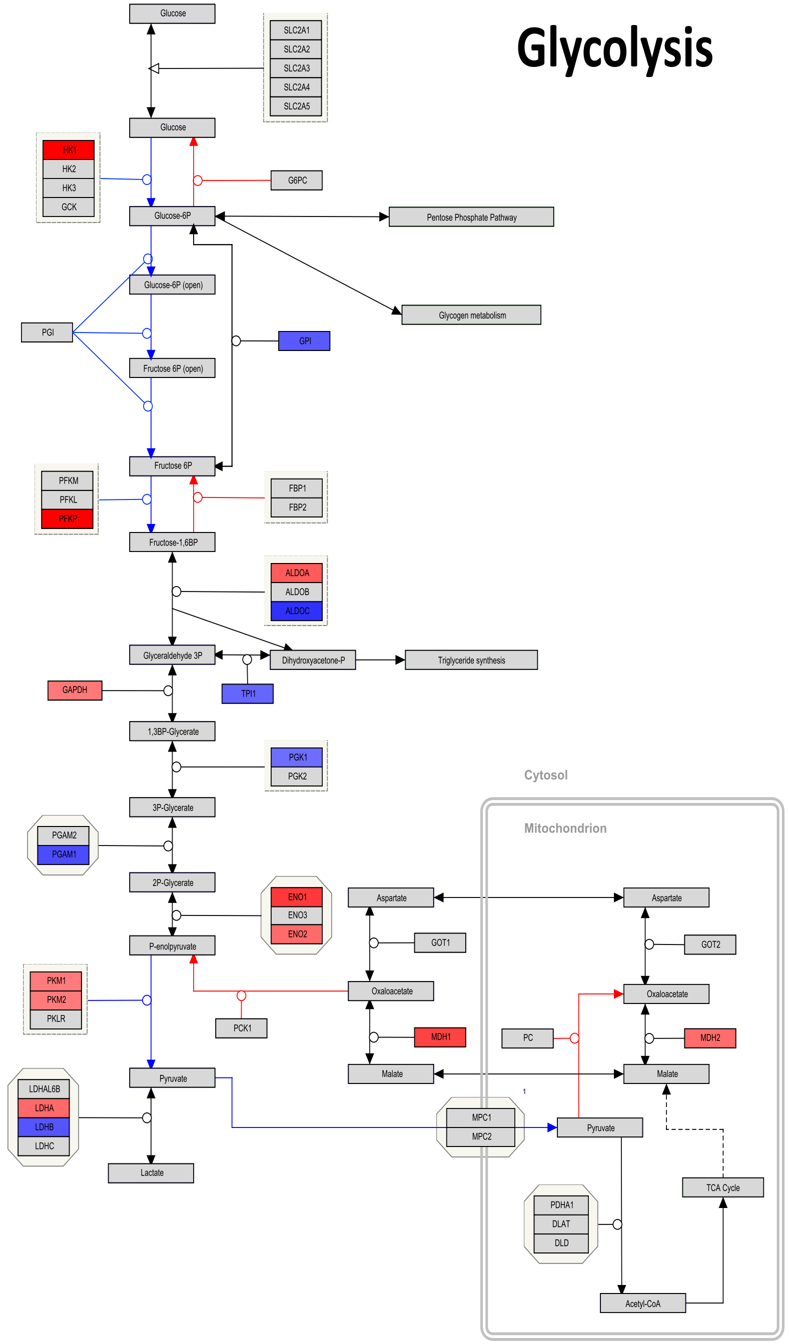

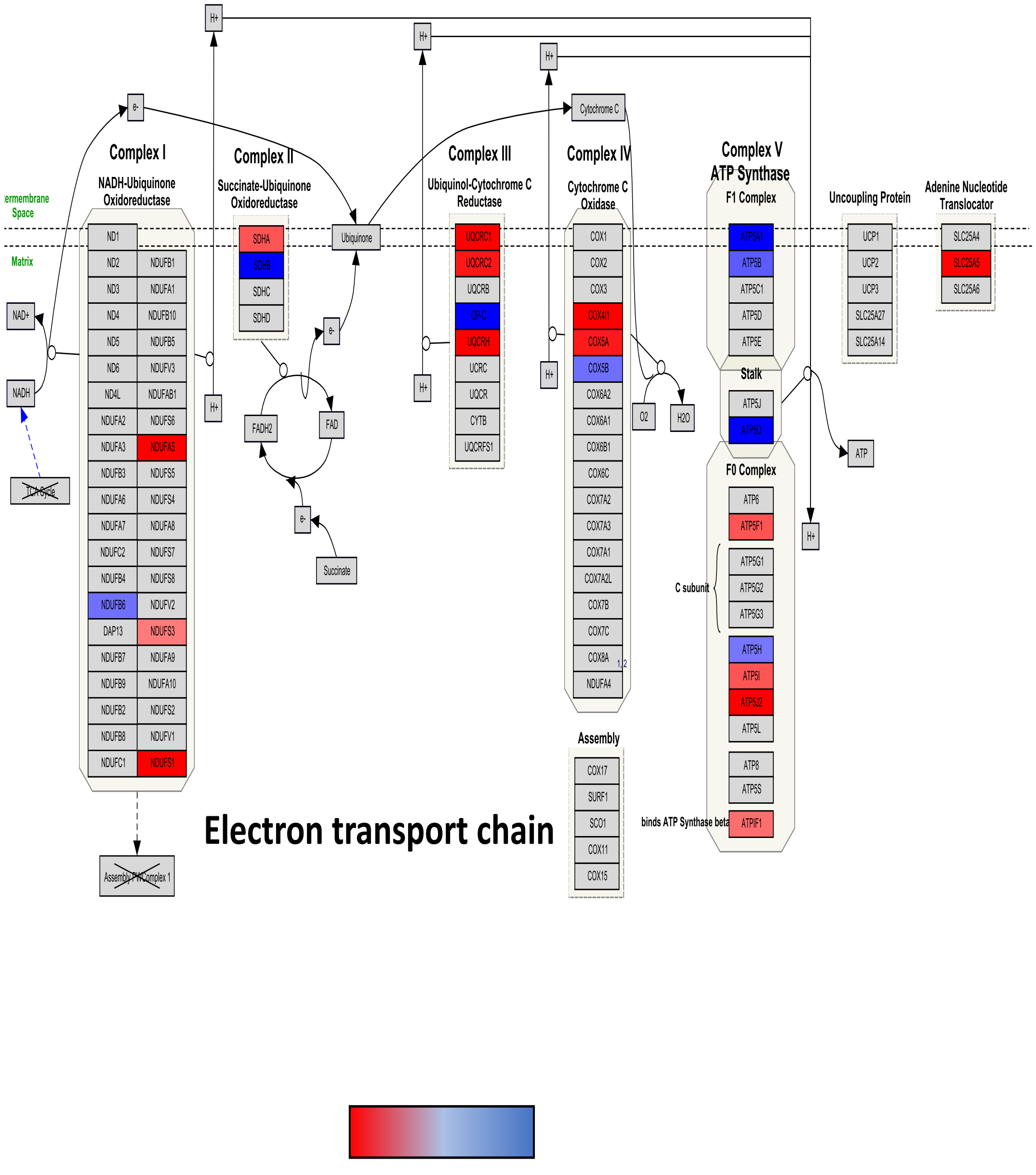

Supplementary Figure 3

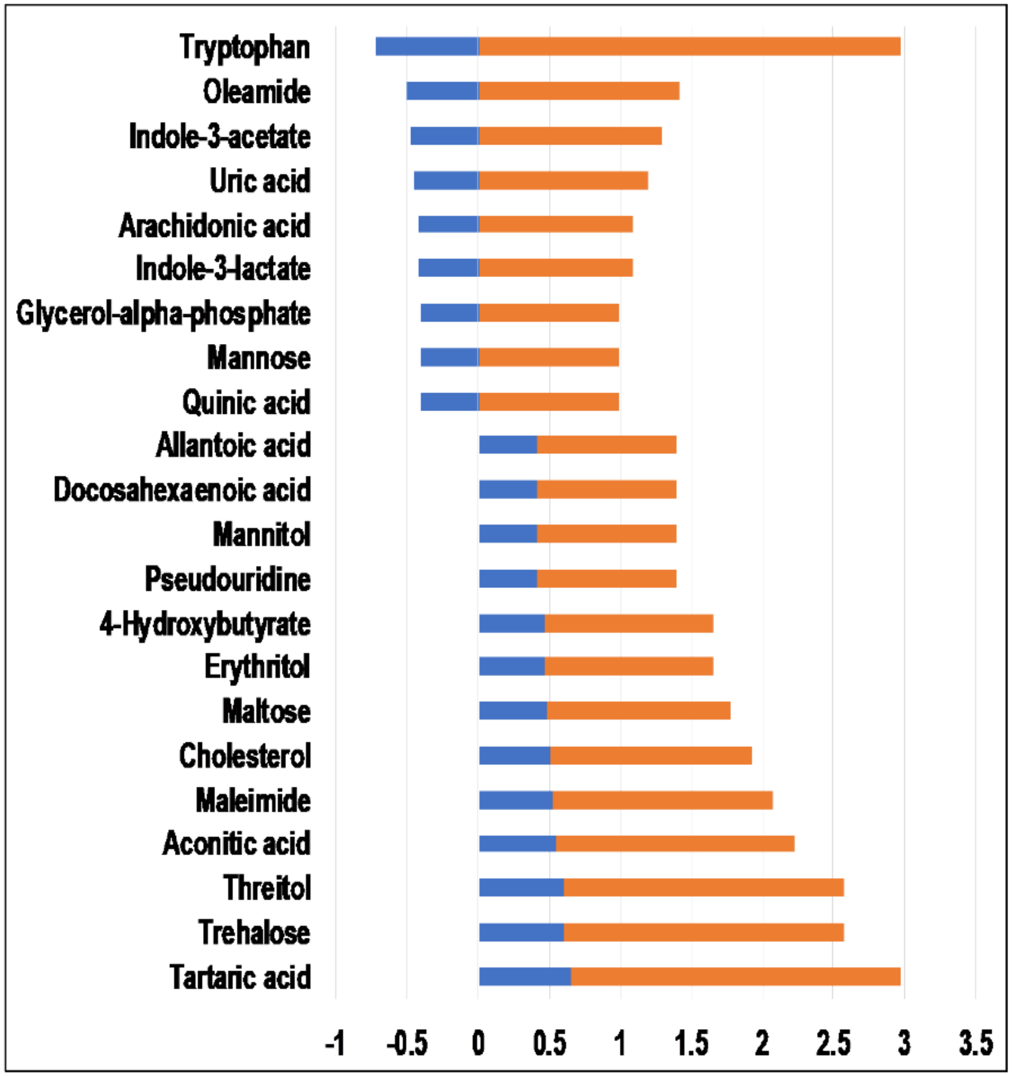

Supplementary Figure 4
